# Size measurements of hepatocellular carcinoma: comparisons between contrast and two-dimensional ultrasound

**DOI:** 10.1186/s12876-020-01535-1

**Published:** 2020-11-19

**Authors:** Fei Chen, Fei Wang, Si Sun, Mei Zhu, Zheng Liu

**Affiliations:** 1 Department of Ultrasound, Xinqiao Hospital, Army Medical University, Chongqing, 400037 China; 2 Department of Ultrasound, First Affiliated Hospital of Kunming Medical University, Yunnan, 650031 China

**Keywords:** Hepatocellular carcinoma, Size measurement, Two-dimensional ultrasound, Contrast enhanced ultrasound

## Abstract

**Background:**

Ultrasound (US) imaging is known to underestimate tumor size in clinical. This study is aimed to compare the size measurements of hepatocellular carcinoma (HCC) in three US imaging modalities, i.e. two-dimensional (2D) imaging, the arterial phase (AP) and the late phase (LP) imaging of contrast-enhanced US (CEUS).

**Methods:**

Fifty-eight clinically proved HCC patients were included. The 2D and CEUS imaging were performed with Siemens S2000, Philips iu22 and BioSound Twice. 2.5 mL of SonoVue® was injected for every CEUS performance. Two physicians measured the maximal longitudinal and the transverse diameters of the tumors in 2D, the AP and the LP of CEUS from one image section. The three measurements were compared by paired *t* test.

**Results:**

The mean longitudinal diameter of HCC appeared to be maximal in the AP (4.73 ± 2.04 cm) of CEUS and minimal in the LP (3.98 ± 1.99 cm) of CEUS. The 2D diameter (4.26 ± 2.07 cm) was in the middle between two CEUS measurements. There were significant differences between any two measurements.

**Conclusion:**

There is size difference between the three kinds of HCC measurement. It appeared to be maximal in the AP of CEUS and minimal in the LP. The 2D diameter was in the middle.

## Background

Hepatocellular carcinoma (HCC) is one of the most common malignancies in clinical. The annual number of new cases of HCC worldwide is over one million, making it the 5th most common cancer worldwide [1, 2] and the third leading cause of cancer-related death [3, 4]. HCC is featured with rapid progress, poor prognosis and high mortality. The size of HCC usually determines the tumor staging and the treatment selections, i.e. either interventional ablation or operational resection [5, 6]. Therefore, the size measurement of HCC is an essential issue for medical imaging, especially the ultrasound (US) imaging which acts as the primary imaging method [7, 8]. However, HCC size is usually underestimated in two-dimensional ultrasound (2DUS) imaging when compared to its surgical size in the experience of our clinical practice, although we didn’t find the related references.

HCC is also a hypervascular tumor with proliferous feeding vessels originated from the hepatic arteries or collateral arteries. In contrast-enhanced US (CEUS), 96% of HCC exhibited a homogeneous or heterogeneous hyperenhancement in the arterial phase (AP), then 2 min later almost all of the hyperenhancement showed a rapid washout and turned to be hypoenhanced in the late phase (LP) [9]. So, CEUS provides another two dynamic contrast phases, AP and LP in which HCC can be well delineated and measured.

Currently, there are no guideline for US measurement of HCC. HCC size is measured mainly based on 2DUS. We presume that HCC size of 2DUS may differ from AP and LP of CEUS. This study is to compare the differences of HCC size between 2DUS, AP and LP of CEUS. The study would be of clinical significance for further accurate size measurement of HCC.

## Methods

### Patients

Between June 2013 and July 2015, fifty-eight patients who underwent 2DUS and CEUS examinations in two hospitals were retrospectively reviewed in this study. The study was approved by the institutional review boards of the two hospitals. They were compared for confirming the size measurements of hepatocellular carcinoma in three US imaging modalities.

All 58 patients (50 males and 8 females with the mean age of 52 ± 10 years, ranged from 33 to 80 years) who suffered from HCCs were enrolled. Thirty-five patients had infected by hepatitis B virus with positive HBV surface antigen (HBsAg). Thirty-two patients had a history of liver cirrhosis. CA-199 elevation (> 37U/mL) was found in twenty-three patients and Alpha-fetoprotein (AFP) was elevated (> 200 mg/mL) in forty-eight subjects. Seven HCCs were proved by biopsy. Nine patients had mild ascites. Twenty-two patients drank alcohol every day, nine of them were addicted to alcohol. Six patients had a history of hypertension. Three patients had a history of diabetes. The clinical diagnoses of HCC were made by AFP elevation, single characteristic dynamic imaging study or dynamic imaging studies such as CEUS, imaging-guided biopsy of the mass [10–13].

Eighteen patients had multiple lesions (2 lesions in 11 patients, and more than 3 lesions in 7 patients) and forty patients had a solitary lesion in each. All lesions had not been treated.

### 2DUS and CEUS examinations

All 2DUS and CEUS examinations were performed with two diagnostic US systems. Fourteen patients were examined with a Philips iU22 (Philips Healthcare Solutions, Bothell, WA) equipped with a C5-1 convex transducer (frequency range 1.0–4.0 MHz) and pulse inversion harmonic imaging. Forty-four patients were imaged with an Esaote MyLab Twice (Esaote S.p.A., Italy) equipped with a CA541 convex transducer and CnTI™ Contrast Tuned Imaging. The US contrast agent used in the study was SonoVue® (Bracco S.p.A., Milan, Italy), a sulphur hexafluoride-filled microbubble covered by a phospholipid shell.

All the 2DUS and CEUS were performed by experienced physicians with 5 years’ experience in liver imaging. The performing physicians were not blinded to on the clinical information and other imaging results. In 2DUS examination of HCC, a clear and large lesion section was usually selected for CEUS. During CEUS examination, dual frame contrast imaging (2DUS and CEUS) was chosen and the image was fixed except the respiratory movement. In the patients with multiple lesions, only the largest lesion in 2DUS was selected for CEUS evaluation. Then, 2.5 mL SonoVue® was administered via the antecubital vein in a bolus fashion (within 1–2 s) followed by a flush of 5 mL of saline by using a 20-gauge cannula. The dynamic cine clips of CEUS were acquired in the AP (0–30 s after injection) and in the LP (120–240 s after injection).

### Size measurement

In retrospective data review, one physician selected a peak enhancement still image in the AP and another image in the LP (peak-enhanced liver) directly on the machines for blind reading. The two images from both phases should match in tumor shape and vicinity. Then, another two physicians who were blinded to the patient clinical information individually measured the maximal longitudinal and transverse diameters of the tumors in 2DUS, AP and LP respectively. The longitudinal and the transverse diameters should almost vertical to each other.

### Statistical analysis

The statistical analyses were performed using the Windows SPSS 20.0 software. For the possible difference of the HCC size read by the two physicians, interobserver agreement was assessed by paired *t* test if the data values obey normal distribution or by Wilcoxon Singned ranks test if the data do not obey normal distribution. The difference between any two of the three HCC measurements was used by paired *t* test (all the data values obeyed normal distribution). *P* value less than 0.05 was considered to be statistically significant.

## Results

The differences of the transverse diameters in 2D mode and the longitudinal diameters in late phase between two readers obeyed the normal distribution, so paired *t* test was used. Wilcoxon Singned ranks test was applied in the remain that not obeyed the normal distribution. Interobserver agreement between the two readers for the tumor size measurement was excellent (Table 1). There were no significant differences in tumor diameter measurement between the two readers (*P* > 0.05).

**Table 1:** Interobserver agreement between two readers for the tumor size measurement

	2DUS		AP		LP	
	LD	TD	LD	TD	LD	TD
Mean ± SD or mean rank	29.86, 28.91	0.02 ± 0.14	28.13, 31.07	29.34, 29.62	0.02 ± 0.10	27.59, 31.16
Z or *t*	− 1.701	0.860	− 0.128	− 0.946	1.521	− 0.856
*p*	0.089	0.394	0.898	0.344	0.134	0.392

*LD* longitudinal diameter, *TD* transvers diameter

^*^P < 0.05 indicates a significant difference between two readers

**Fig. 1:**
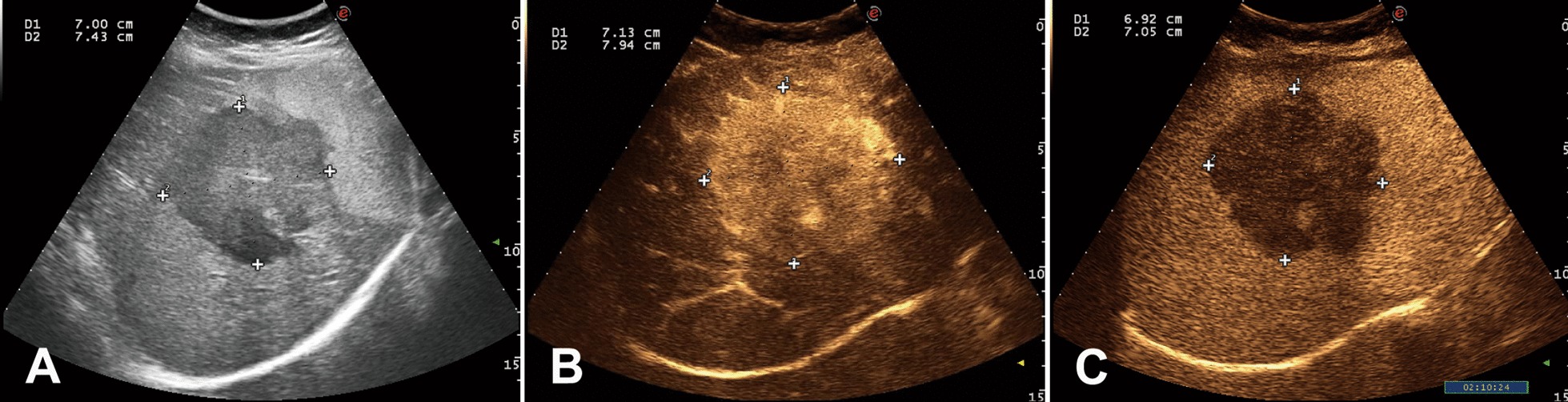
The measurements of 2D, AP and LP diameters of a HCC case on the same imaging section (Data on left upper corner, D1 = longitudinal diameter; D2 = transverse diameter). **a** 2DUS measurements of the tumor. **b** Obviously enlarged measurement of the tumor in AP. **c** Decreased measurement of the tumor in LP

**Fig. 2:**
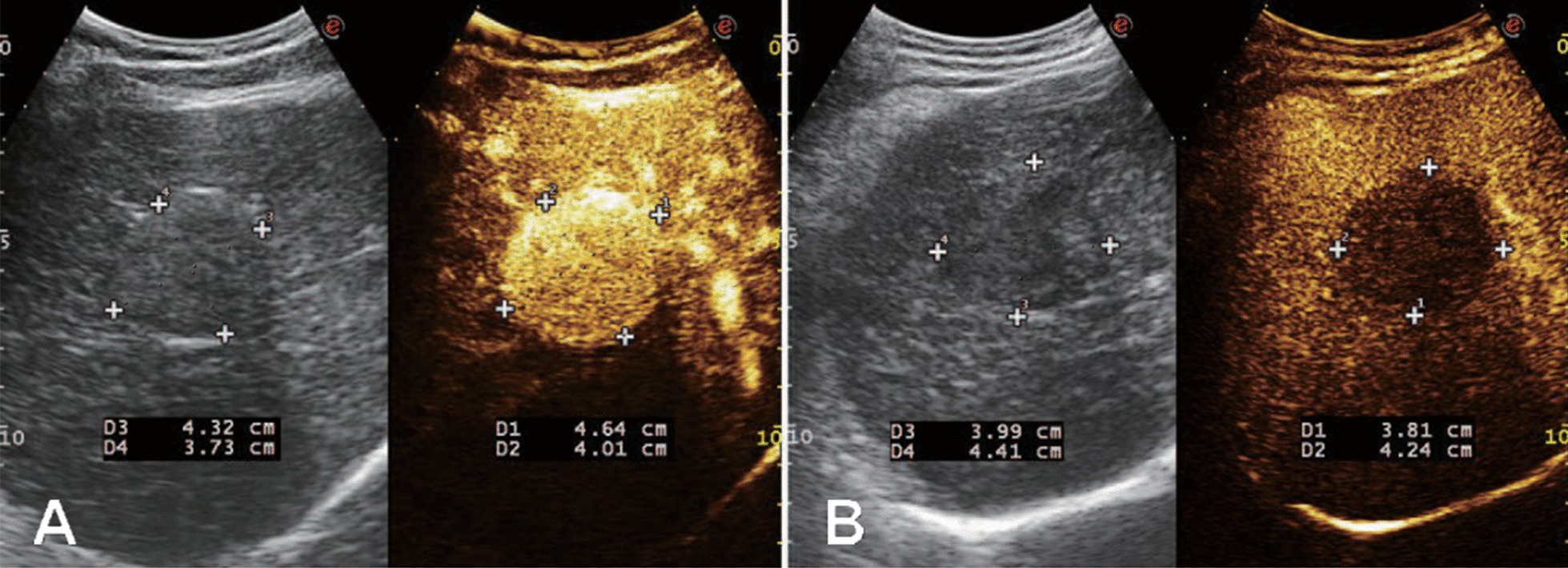
The measurements of diameters from another two cases. **a** The dual frame of 2D (left) and AP (right) measurements showing slightly enlarged AP diameters in the first case. **b** The dual frame of the second case displaying slightly decreased measurements in LP (right) when comparing that of 2D (left)

**Fig. 3:**
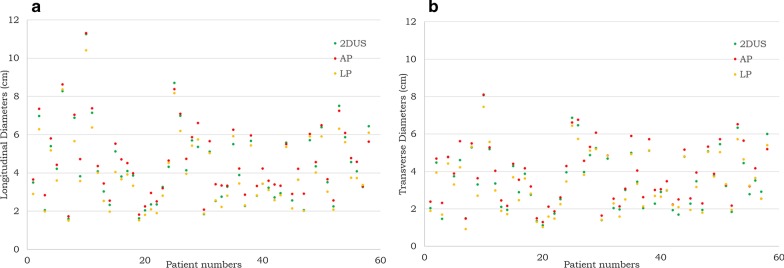
Scatter plot of the longitudinal diameters (**a**) and transverse diameters (**b**) from all 58 cases. In most of the cases, AP diameters appeared to the maximal followed by 2D diameters, and then LP diameters

All the maximal longitudinal and transverse diameters of the tumors in 2D mode, AP and LP measured by the two readers obeyed the normal distribution. All the difference values between 2D mode and AP, 2D mode and LP, AP and LP obeyed the normal distribution (*P* > 0.05). All the measurements of the longitudinal and transverse diameters were compared by a paired *t* test. The mean value of measurement by two physicians reflected the order of the size through different US modalities (Table 2). The maximal longitudinal and transverse diameters of the tumors in 2D mode were 9.7% and 9.8% smaller than that of the AP (Figs. 1a, b, 2a), and were 5.6% and 5.4% larger than that of LP (Figs. 1a, c, 2b). However, the longitudinal and transverse diameters of the tumors in AP were 14.8% and 14.7% larger than that of LP respectively (Figs. 1b, c, 2a, b). In the scatter plot (Fig. 3a, b), AP diameter at the highest value, then followed by the 2DUS, LP diameter at the lowest value in most of HCC cases.

**Table 2:** Mean values of diameter measurements of two readers

	2DUS (Mean ± SD)		AP (Mean ± SD)		LP (Mean ± SD)	
	LD	TD	LD	TD	LD	TD
Reader 1	4.26 ± 2.07	3.51 ± 1.70	4.72 ± 2.03*	3.89 ± 1.64*	4.02 ± 2.02^*#*^	3.32 ± 1.58^*#*^
Reader 2	4.26 ± 2.07	3.49 ± 1.69	4.70 ± 2.07*	3.87 ± 1.63*	4.00 ± 2.01^*#*^	3.33 ± 1.58^*#*^

*LD* longitudinal diameter, *TD* transvers diameter

^*^Indicates significant larger than that of the corresponding 2DUS diameters

^*#*^Indicates significant smaller than that of the corresponding 2DUS diameters

## Discussion

Tumor angiogenesis is vital to tumor growth, invasion and metastasis [14, 15]. The results of angiogenesis lead to the formation of proliferous vasculature including the surrounding feeding vessels. A few studies have shown that the growth and progression of tumor are greatly associated with an increase in feeding vessels through the process of angiogenesis [16]. The feeding vessels of solid tumors usually originate from two sources. One source is the existing vascular network of the tumor body, morphologically mature and active blood vessels [17, 18]. Another source is angiogenetic microvasculature formed through the process of tumor vascularization [19, 20]. The angiogenetic microvasculature is characteristic with irregular vascular size, shape and branching pattern. HCC is typical hypervascular tumor with abundant network of feeding vessels, most of HCC demonstrated a homogeneous or heterogeneous hyperenhancement in AP of CEUS. A few studies have shown that microvascular density, vascular invasion are the strong predictors of tumor outcome and recurrence for HCC [21–23]. Therefore, the entire network of feeding vessels should be resected or ablated during surgical resection or interventional ablation therapy. Incomplete treatment may result in some survival vessels and residual tumors.

Conventional 2DUS is unable to image the vascular network of tumor, yet color Doppler flow imaging (CDFI) can only reveal the main branches of the network because of the poor sensitivity to low-speed blood flow. In small HCCs, the sensitivity of CDFI in showing abnormal arterial neovascularity is low, and flow can be demonstrated in less than 50% of the lesions [24, 25].

CEUS is relatively new US imaging modality that can show the dynamic contrast perfusion of tumor body including capillary. In AP, CEUS is able to demonstrate an integrated wash-in hyperenhancement of intratumoral and peritumoral tumor vessels, while in LP the hyperenhancement quickly wash out and HCC becomes hypoenhanced [26]. This dynamic phase changes provide another two opportunities to measure HCC besides 2DUS.

In this study, we found that the HCC sizes of 2D, AP and LP are significantly different. The maximal longitudinal and transverse diameters of AP were 9.7% and 9.8% larger than that of 2D, and were 14.8% and 14.7% larger than that of LP. Likewise, the 2D longitudinal and transverse diameters were 5.6% and 5.4% larger than that of LP. The interobserver agreement between two readers was excellent, so there were no significant differences on size measurement between the readers. AP diameter of HCC appeared to be maximal followed by 2D diameter, and LP diameter was the minimal. Despite the order of three sizes was clearly seen in most of HCC cases in the scatter plot (Fig. 3), we also found the data dispersion of individual case vary greatly, indicating the heterogenicity of tumor angiogenesis.

We prefer to use routine 2DUS for HCC general screening high-risk subjects, because it is more convenient, time-saving and less costly. For HCC patients requiring surgical resection or interventional therapies, especially radiofrequency ablation, we could recommend AP as more precise measurement. It is obvious that AP diameter includes tumor vascular construction not only intratumoral but also peritumoral vascular invasion. So, it is understandable that AP diameter is larger than 2D diameter which does not contain peritumoral vascular construction. Considering the importance of peritumoral vascular invasion in HCC prognosis and recurrence [27, 28], AP diameter should be selected for therapeutic reference.

In CEUS, LP usually provides the best contrast resolution for HCC imaging or size measurement. However, LP diameter was the minimal when compared to AP and 2D diameters in this study. LP diameter may underestimate HCC size probably because some well-differentiated HCC tend to iso-enhanced and incomplete washout in LP. The iso-enhancement may shrink the sizing range of HCC. The quick and significant washout is observed more frequently in HCC with poorer grades of differentiation than in well-differentiated HCC [29–31]. Thus, it may be inappropriate to use LP diameter for treatment reference.

There are some limitations in this study. Firstly, the study was designed and limited to only HCC population. Secondly, it is obvious that AP cannot substitute CT or MRI for tumor size measurement. The sections in ultrasound imaging are usually flexible and acoustic window dependent, not as standardized as in CT or MRI. The US imaging cannot match CT or MRI in section, therefor the comparison is not appropriate. So, we think that AP can only correct the bias of underestimation in ultrasound measurement. Thirdly, the HCC size did not compare with gross pathological size, which is the gold standard. This research is a preliminary, retrospective study not a randomized controlled trial. Further large sample study is needed to prove the results.

## Conclusion

In conclusion, there is size difference between the three kinds of HCC measurement in this pilot study. AP diameter appeared to be maximal and it is larger than that of 2D or LP diameters.

## Data Availability

The datasets used and analysed during the current study are available from the corresponding author on reasonable request.

## Abbreviations

HCC: Hepatocellular carcinoma
CEUS: Contrast-enhanced ultrasound
2DUS: Two-dimensional ultrasound
TUS: Therapeutic ultrasound
US: Ultrasound
AP: Arterial phase
LP: Late phase
